# Determination of Ghrelin Structure in the Barfin Flounder (*Verasper moseri*) and Involvement of Ingested Fatty Acids in Ghrelin Acylation

**DOI:** 10.3389/fendo.2013.00117

**Published:** 2013-09-03

**Authors:** Hiroyuki Kaiya, Tadashi Andoh, Takashi Ichikawa, Noriko Amiya, Kouhei Matsuda, Kenji Kangawa, Mikiya Miyazato

**Affiliations:** ^1^Department of Biochemistry, National Cerebral and Cardiovascular Center, Suita, Japan; ^2^Seikai National Fisheries Research Institute, Fisheries Research Agency, Nagasaki, Japan; ^3^Hokkaido National Fisheries Research Institute, Fisheries Research Agency, Akkeshi, Japan; ^4^School of Marine Biosciences, Kitasato University, Sagamihara, Japan; ^5^Laboratory of Regulatory Biology, Graduate School of Science and Engineering, University of Toyama, Toyama, Japan; ^6^National Cerebral and Cardiovascular Center, Suita, Japan

**Keywords:** acyl modification, barfin flounder, cDNA cloning, feed, fatty acid, ghrelin, ingestion

## Abstract

Ghrelin is a peptide hormone that is acylated with a fatty acid, usually *n*-octanoic acid, at the third amino acid (aa) residue (usually a serine or threonine), and this acylation is known to be essential for ghrelin activity not only in mammals but also in non-mammals, such as fish. However, the modification mechanisms of ghrelin modification in fish are not known. In this study, we elucidated the structure of ghrelin in a teleost, the barfin flounder (*Verasper moseri*), and determined whether ingested free fatty acids of various chain lengths participated in ghrelin acylation. Complementary DNA cloning revealed the barfin flounder prepro-ghrelin to be a 106-aa peptide and the mature ghrelin to be a 20-aa peptide (GSSFLSPSHKPPNKGKPPRA). However, purification of ghrelin peptides from stomach extracts demonstrated that the major form of the hormone was a 19-aa decanoylated peptide [GSS(C10:0)FLSPSHKPPNKGKPPR] missing the last alanine of the 20-aa peptide. Ingestion of feed enriched with *n*-heptanoic acid (C7), *n*-octanoic acid (C8), or *n*-non-anoic acid (C9) changed the modification status of the peptide: ingestion of C8 or C9 increased the amount of C8:0 or C9:0 19-aa ghrelin, respectively, but no C7:0 ghrelin was isolated after ingestion of C7. These results indicate that ingested free fatty acids are substrates for ghrelin acylation in the barfin flounder, but the types of free fatty acids utilized as substrates may be limited.

## Introduction

Ghrelin, a peptide hormone produced in gastric ghrelin cells (called X/A-like cells in rats and P/D cells in humans), is acylated at the third amino acid (aa) residue, usually a serine or threonine (1, 2). Acylation is essential for binding of the hormone to its receptor [growth hormone secretagogue receptor type 1a (GHS-R1a)] and for eliciting its activities (1). Acylation, usually with *n*-octanoic acid (C8), occurs post-transcriptionally; but des-acyl ghrelin, which lacks a fatty acid side chain and does not activate GHS-R1a, is also produced in the same ghrelin cells. Ghrelin acylation is catalyzed by ghrelin *O*-acyltransferase [GOAT; (3, 4)], a member of the membrane-bound *O*-acyltransferase superfamily. The expression pattern of the enzyme overlaps that of ghrelin in gastric mucosal cells, suggesting that these cells play a key role in ghrelin synthesis (5, 6).

Ghrelin can be acylated by fatty acids other than C8, as is the case in cats, rats, and humans (7, 8). Several studies have investigated the source of substrates for ghrelin acylation. Nishi et al. (9) demonstrated that in mice, dietary lipids (free fatty acids or medium-chain triacylglycerols) contribute to ghrelin acylation. Subsequent studies demonstrated that these triacylglycerols are substrates for GOAT-mediated acylation of ghrelin (3), and GOAT plays an important role in the acylation [(10); see also the review by Shlimun and Unniappan (11)].

Ghrelin is also present in a wide variety of non-mammalian vertebrates (12, 13), and ghrelins acylated by fatty acids other than C8 have also been identified in various animals from birds to elasmobranchs, including chickens (14), red-eared slider turtles (15), bullfrogs (2), rainbow trout (16), Japanese eels (17), Mozambique tilapia (18), channel catfish (19), goldfish (20), and sharks (21). In Mozambique tilapia, decanoylated ghrelin (C10) is the major form of the peptide, and the physiological actions of C8- and C10-ghrelin are different (22, 23), suggesting that the type of fatty acid modification is an important determinant of ghrelin’s actions.

The source of fatty acids for ghrelin acylation has been investigated in neonatal chicks (24): oral or intraperitoneal administration of *n*-octanoic acid increases the amount of C8-ghrelin in the proventriculus (a glandular portion of the stomach of chickens) and in plasma. However, whether such a mechanism for ghrelin acylation exists in other non-mammalian vertebrates is not known.

Our goal in this study was to determine whether exogenous free fatty acids contribute to ghrelin acylation in fish. We used a cultured flatfish, the barfin flounder (*Verasper moseri*). This species is distributed over the Hokkaido Pacific coast. At one point, hauls of the fish had decreased so substantially that it was referred to as “the rarely seen fish,” but catches have been increasing as a result of the release of aquacultured fish. First, we determined the structure of ghrelin in the barfin flounder by means of both cDNA cloning and peptide purification. Then we determined the modification status of ghrelin peptides purified from the stomach extracts of fish that were given feed enriched in *n*-heptanoic (enanthic) acid (C7), *n*-octanoic (caprylic) acid (C8), or *n*-nonanoic acid (pelargonic) acid (C9). The results of the feeding experiment indicate that ingested free fatty acids were substrates for ghrelin acylation in the barfin flounder, as has been demonstrated in mice and chickens, but the type of fatty acid utilized as substrate is likely to be limited.

## Materials and Methods

### Cloning of ghrelin cDNA in barfin flounder

Adult male barfin flounder (*V. moseri*) were obtained from the Iwate Fisheries Technology Center (Kamaishi, Iwate, Japan). The fish were reared under natural photoperiod and temperature conditions in seawater. Stomach and intestine were dissected out of fish anesthetized by immersion in 0.05% 2-phenoxyethanol, and the organs were frozen in liquid nitrogen and stored at −85°C until use. Total RNA was obtained from stomach and intestine of two individuals by means of a QIAcube (QIAGEN, Hilden, Germany) and an RNeasy Mini Kit (QIAGEN); and poly(A)^+^ RNA was purified using an Oligotex dT30 Super (TaKaRa Bio Inc., Shiga, Japan). First-strand cDNA was synthesized from 132 ng of poly(A)^+^ RNA (half from the pooled stomach RNA and the other half from the pooled intestinal RNA) by means of a First-Strand cDNA Synthesis Kit (Amersham Pharmacia Biotech, Buckinghamshire). Reverse transcription was performed at 25°C for 10 min, at 42°C for 1 h, and at 51°C 30 min, followed by 5 min at 99°C with an adaptor primer (ATTCTAGAGGCCGAGGCGGCCGACATG-d(T) _30_-VN).

3′-Rapid amplification of cDNA ends (RACE) PCR was conducted using a degenerate sense primer based on known ghrelin sequences (5′-TNG GNM GNC ARA CNA TGG ARG-3′) and the adaptor primer described above with preheating at 94°C for 2 min followed by 35 cycles at 94°C for 40 s, 60°C for 40 s, and 72°C for 1 min in a thermocycler (Whatman Biometra, Göttingen, Germany). The PCR products were electrophoresed on 3% agarose gel (Agarose S, Nippon Gene, Tokyo, Japan) containing 0.005% ethidium bromide. The cDNA fragment of the expected size was cut out, purified by means of a Wizard SV Gel and PCR Clean-Up System (Promega, Madison, WI, USA), and ligated into the pT7Blue T-Vector (Novagen, San Diego, CA, USA). Plasmid DNA containing the expected insert was extracted from bacterial culture with a High Pure Plasmid Isolation Kit (Roche Diagnostics, Mannheim, Germany). The sequencing reaction was performed with a BigDye Terminator Cycle Sequencing Kit (Applied Biosystems, Foster City, CA, USA).

5′-RACE PCR was performed with gene-specific antisense primers based on the sequence determined by 3′-RACE PCR, as well as a 5′-primer. Primary and nested PCRs were performed with GSP-BF-ghrelin R1 (5′-GGC AAC TGA CAC TTT TAC TC-3′) and GSP-BF-ghrelin R2 (5′-TTC AAT GAT CAC TAT CTA ATG-3′) primers, respectively. To confirm the total nucleotide sequence, we amplified the full-length cDNA with a sense primer (BarfinGHRL-full-s: 5′-GTT TAA GGT CCA CTA ACT CAG GGG-3′) and a 3′-primer.

### Purification of ghrelin from stomach extracts of barfin flounder fed normal feed

Frozen stomach (12.4 g) collected from a fish that ate normal feed, as described in the next section, was used for the starting material.

To follow ghrelin activity during the purification process, we measured changes in intracellular Ca^2+^ concentration in CHO-GHSR62 cells, a cell line stably expressing rat GHS-R1a (1). Samples (1/40 to 1/200 volume of collected fraction) were used for this assay. The cells were plated onto a 96-well black plate at a density of 5 × 10^4^ cells per well. Twenty hours after plating, the culture medium was aspirated, and 100 μl of fluorescent dye solution containing 4.4 μM Fluo-4AM (Life Technologies, Foster City, CA, USA), 1% fetal calf serum, and 1% PowerLoad solution (Life Technologies) in a working buffer was loaded onto each well. After the plate was incubated for 1 h at 37°C, the cells were washed three times with the working buffer. Sample was dissolved with the working buffer containing 0.001% Triton X-100, and an intracellular Ca^2+^ mobilization assay was performed automatically with a fluorometric imaging plate reader (FLIPR Tetra, Molecular Devices, Menlo Park, CA, USA).

Stomach tissues were boiled for 10 min with five volumes of Milli-Q water, cooled, and then acidified with 1 M acetic acid. The tissues were homogenized, and the supernatant was obtained by centrifugation at 13,400 × *g* for 30 min. The crude acidic extracts were loaded onto a Sep-Pak Plus C18 cartridge (Waters, Milford, MA, USA), and adsorbed peptides were eluted first with 30% and then with 60% acetonitrile containing 0.1% trifluoroacetic acid (TFA). The solution eluted with 60% acetonitrile containing 0.1% TFA was subjected to cation-exchange chromatography (SP-Sephadex C-25, H^+^-form, GE Healthcare Bio-Science Corp., Piscataway, NJ, USA), and successive elution with 1 M acetic acid, 2 M pyridine, and 2 M pyridine/acetic acid (pH 5.0) yielded three fractions: SP-I, SP-II, and SP-III, respectively. The strongly basic peptide-enriched SP-III fraction was purified with a Sep-Pak Plus C18 cartridge, and the evaporated sample was subjected to gel-filtration high-performance liquid chromatography (HPLC) on a TSKgel G2000 SWxL column (21.5 mm × 300 mm, custom order, Tosoh, Tokyo, Japan) with 35% acetonitrile containing 0.1% TFA as the eluent at a flow rate of 2 ml/min. Fractions showing ghrelin activity were lyophilized, loaded onto a carboxymethyl (CM) ion-exchange HPLC column (TSKgel CM-2SW, 4.6 mm × 250 mm, Tosoh), and eluted at 1 ml/min with a four-step solvent gradient consisting of mixtures of solution A (9:1 mixture of 10 mM HCOONH_4_ and acetonitrile, pH 4.8) and solution B (9:1 mixture of 1 M HCOONH_4_ and acetonitrile, pH 4.8) as follows: increase from 0 to 25% B over 5 min (step 1), increase from 25 to 65% B over 60 min (step 2), increase from 65 to 100% B over 5 min (step 3), and hold at 100% B for 10 min (step 4). Fractions (1 ml/tube) were collected every minute for 80 min. Fractions showing ghrelin activity were separated by reverse-phase HPLC on a Symmetry300 C18 column (3.9 mm × 150 mm, Waters, Milford, MA, USA); the eluent was a linear gradient of acetonitrile containing 0.1% TFA (10–60% over 40 min; flow rate, 1 ml/min). Fractions (0.5 ml/tube) were collected starting 15 min after injection. Fractions showing ghrelin activity were further purified by reverse-phase HPLC on a diphenyl column (2.1 mm × 150 mm, 219TP5125, GRACE Vydac, ChemcoPlus Scientific Co., Osaka, Japan); the eluent was a linear gradient of acetonitrile containing 0.1% TFA (10–60% over 40 min; flow rate, 0.2 ml/min). The eluate corresponding to each absorbance peak was collected.

The sequences of isolated peptides were analyzed with a protein sequencer (model 494HT, Life Technologies). The molecular weights of all isolated peptides were measured with an AB SCIEX TOF/TOF 5800 system (AB SCIEX, Tokyo, Japan) with α-cyano-4-hydroxycinnamic acid (Sigma-Aldrich Co., St. Louis, MO, USA) as a matrix.

### Feeding experiment with fatty acid-enriched feed

The barfin flounder used in the feeding experiment were bred at Akkeshi Station, Hokkaido National Fisheries Research Institute, Fisheries Research Agency, Japan. Two-year-old flounder were kept in 2000 l aquaria with running seawater controlled at 17.0 ± 0.5°C. Flounder were fed apparent satiation with commercial dry pellets (P-8 for flounders, diameter 1.5 cm, Higashimaru, Kagoshima, Japan) once daily every business day for 2 months until the start of the feeding experiment. Regarding the fatty acid compositions in the diet, there were the data about the long-chain fatty acids but not the data of the middle-chain fatty acids. In the long-chain fatty acids, palmitic acid, oleic acid, icosapentaenoic acid, and docosahexaenoic acid predominated.

We divided the fish into four experimental groups consisting of three fish each: group 1 was fed normal feed, group 2 was fed C7-enriched feed, group 3 was fed C8-enriched feed, and group 4 was fed C9-enriched feed. The total body lengths and body weights (means ± SDs) of each group were 479.8 ± 12.7 mm and 1729.8 ± 222.6 g for group 1, 436.5 ± 43.8 mm and 1356.5 ± 675.6 g for group 2, 417.8 ± 54.8 mm and 1155.5 ± 461.9 g for group 3, and 441.3 ± 11.9 mm and 1217.8 ± 189.4 g for group 4; there were no significant differences in either body length or weight among the groups.

The enriched feeds were prepared by manually pipetting 20 μl of either *n*-heptanoic, *n*-octanoic, or *n*-non-anoic acid onto each feed pellet, and then 20 μl of 10% (v/w) aqueous L-alanine was added dropwise as a feeding attractant. The feed for the control group was treated only with the L-alanine solution. The feeding experiment was started on a Monday, and fish were fed every Monday, Wednesday, and Friday for a total of seven feedings at a rate of 2% of body weight per day; as a result, each fish ingested approximately 140 μl per individual per day of each fatty acid. The experiment was performed twice, and the total number of fish in each group ranged from four to six individuals. Sampling was conducted 24 h after the final feeding (day 16, which was the third Tuesday morning). The fish were anesthetized in 0.01% aqueous ethyl *p*-aminobenzoate (Wako Pure Chemical Industries, Osaka, Japan), and stomach were dissected out, rinsed with saline (0.9% NaCl), cut longitudinally, and frozen. The stomach samples were kept at −80°C until use. The stomach samples collected as described in this section were used for purification of ghrelin (stomach weights: normal feed, 12.4 g; C7-enriched feed, 7.6 g; C8-enriched feed, 10.5 g; C9-enriched feed, 14.5 g) as described above and for profiling of ghrelin in each stomach by reverse-phase HPLC as described below.

### Profiling of barfin flounder ghrelin in stomach samples

Peptide components were extracted from frozen stomach samples, approximately 1 g, from each fish (normal feed, *n* = 4; C7-enriched feed, *n* = 4; C8-enriched feed, *n* = 6; C9-enriched feed, *n* = 6) as described earlier. Each resulting stomach extract (equivalent to 100 mg of tissue) was placed on a Sep-Pak Plus C18 column and eluted with 60% acetonitrile containing 0.1% TFA. The eluate was subjected to reverse-phase HPLC on a Symmetry300 C18 column; the eluent was a linear gradient of acetonitrile containing 0.1% TFA (10–60% over 80 min; flow rate, 1 ml/min). To confirm the elution times of the isolated peptides, we also subjected representative isolated native barfin flounder ghrelin to reverse-phase HPLC under the same conditions used for the stomach extracts. Fractions (1 ml/tube) were collected starting 30 min after injection, and ghrelin activity in the fractions was measured by means of Ca^2+^ mobilization assay with CHO-GHSR62 cells.

### Quantitative PCR for barfin flounder ghrelin mRNA in the stomach

To evaluate the effect of fatty acid ingestion on ghrelin mRNA expression in the stomach, we conducted quantitative PCR for ghrelin. A ghrelin fragment (337 bp) was amplified from stomach cDNA by means of reverse-transcription PCR with a primer pair for ghrelin (BarfinGHRL Q-s: 5′-AGC TGC TGG TTT TTC TAC TCT GTT-3′; BarfinGHRL Q-AS: 5′-AAA GGT AAA TCT GCC ATT CTT GTC-3′). As an internal control, β-actin (629 bp) was quantified with a primer pair (BarfinB-actin-s: 5′-TGA AGT ACC CCA TCG AGC AC-3′; BarfinB-actin-AS: 5′-TAC AGG TCC TTA CGG ATG TC-3′). Quantitative PCR was performed with a LightCycler 480 instrument (Roche Applied Science, Mannheim, Germany) and a QuantiFast SYBR Green PCR Kit (QIAGEN). The amplification conditions were as follows: 95°C for 5 min followed by 40 cycles at 95°C for 10 s and 60°C for 30 s. The reaction mixture consisted of 1× master mix, the two primers (250 nM each), and a template (equivalent to 100 ng of total RNA) prepared by QuantiTect Reverse-Transcription Kit (QIAGEN). For quantification of mRNA copy numbers, a linear regression line was generated using a serially diluted pCRII vector containing each cloned cDNA fragment that was linearized by restriction with *Xba*I.

### Statistical analyses

In the experiment on profiling of ghrelin in stomach extracts, the values for each group were compared to the control values by analysis of variance followed by Fisher’s protected least significant difference test. For comparison of ghrelin mRNA expression in the stomach, non-parametric Mann–Whitney *U* test was applied. Differences were considered significant at *P* < 0.05.

## Results

### Structure of barfin flounder ghrelin

**Figure 1:**
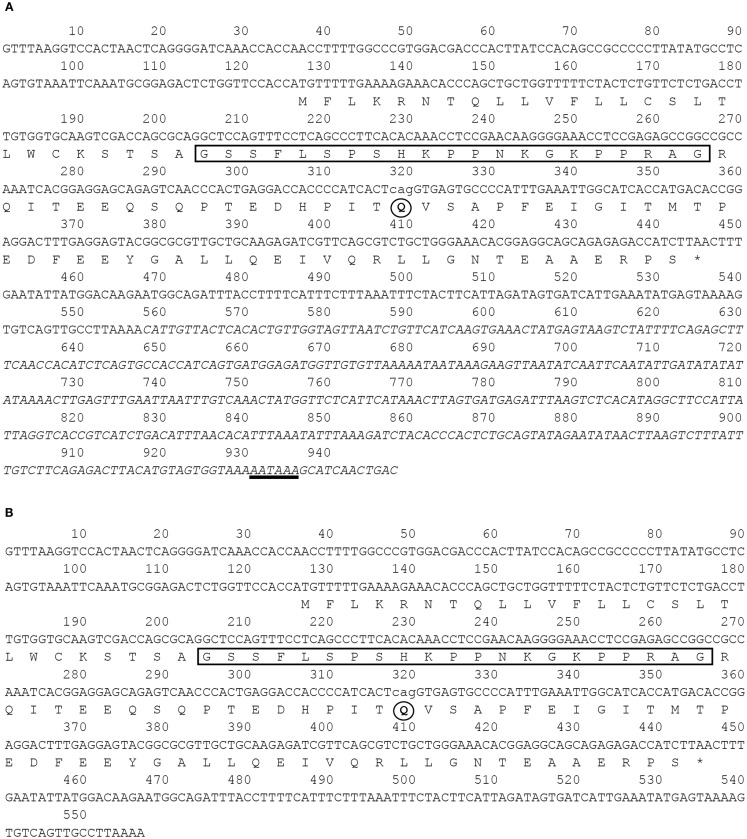
**Nucleotide and deduced amino acid sequences of barfin flounder ghrelins**. The asterisk indicates the termination codon, the box indicates the sequence of the mature ghrelin, and the underlining indicates the polyadenylation signal (AATAAA). Four different cDNAs were obtained: **(A)** a 948-bp cDNA (acc. no. AB823534) and a 945-bp variant missing the codon for Q65 (acc. no. AB824842) and **(B)** a 557-bp cDNA missing the nucleotides italicized in **(A)** (acc. no. AB824843) and a 554-bp cDNA missing the italicized nucleotides and the codon for Q65 (acc. no. AB824844).

**Figure 2:**
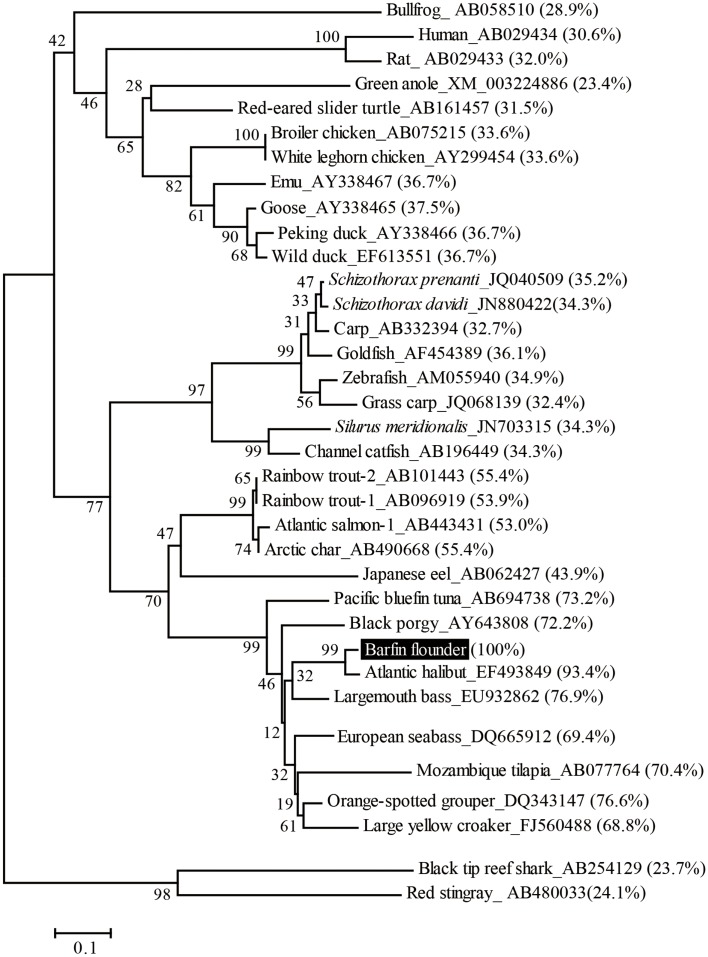
**Phylogenetic analysis of the barfin flounder prepro-ghrelin**. The phylogenetic tree was generated by means of the NJ method of the MEGA4 software package (http://www.megasoftware.net/). The GenBank accession numbers follow the species names, the percentages in parentheses indicate amino acid identities to the barfin flounder prepro-ghrelin, and the numbers at the branch points represent bootstrap values (1000 repetitions).

**Figure 3:**
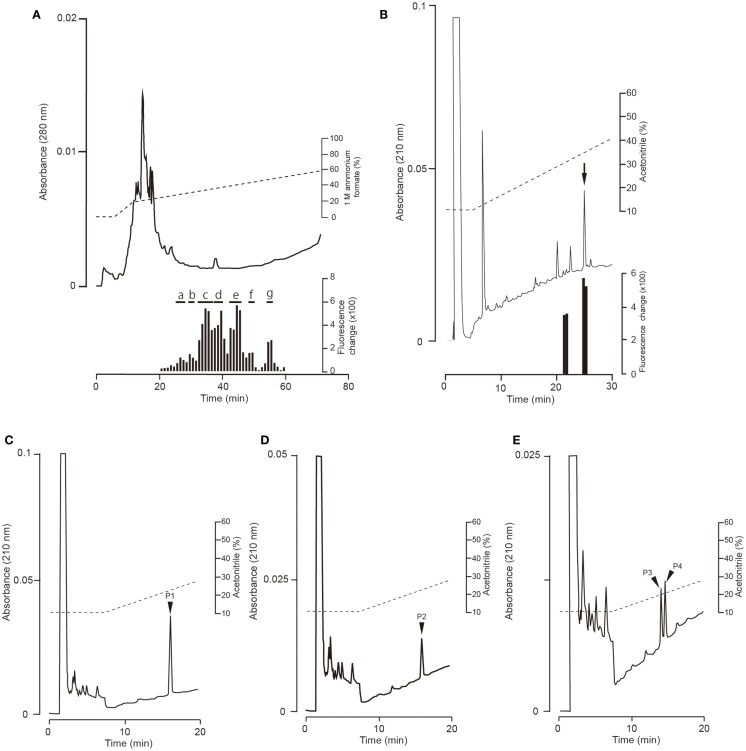
**Representative absorption spectra and activity data obtained at various points during the purification of ghrelin from the stomach of barfin flounder fed normal feed**. **(A)** Carboxyl methyl ion-exchange HPLC. Black bars indicate ghrelin activity measured using CHO cells expressing rat GHS-R1a. The fractions were divided into groups a–g on the basis of ghrelin activity. **(B)** Reverse-phase HPLC of group “e” on a symmetry 300 C18 column. The arrow indicates the major peak. **(C)** Reverse-phase HPLC of group “e” on a diphenyl column. The peak labeled P1 corresponds to the peak indicated by the arrow in **(B)**; this peak corresponds to the peptide obtained in the highest yield from the stomach of fish fed normal feed. **(D)** The peptide (P2) obtained in the second highest yield (isolated from group “g”). **(E)** The peptides (P3 and P4) obtained in the third and fourth highest yields (isolated from group “c”).

**Figure 4:**
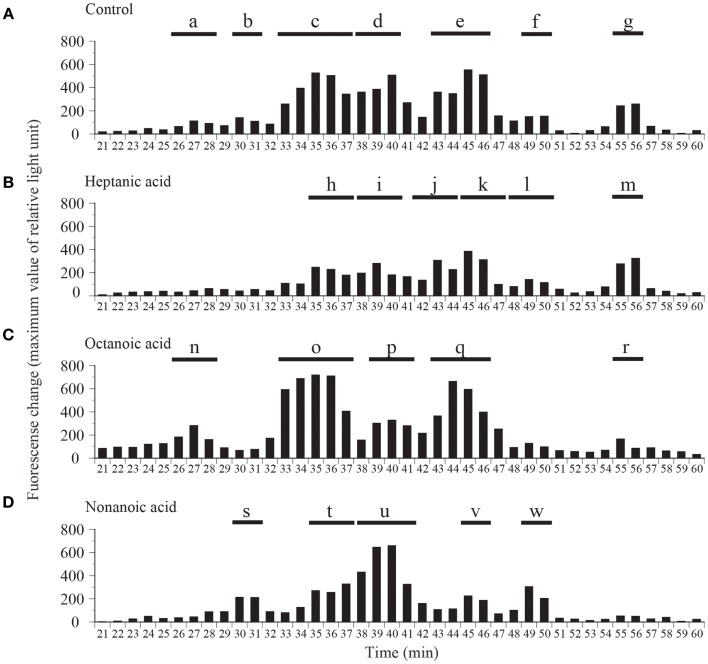
**Distribution of ghrelin activity in fractions separated by ion-exchange HPLC of samples obtained from fish fed (A) normal feed, (B) *n*-heptanoic acid (C7)-enriched feed, (C) *n*-octanoic acid (C8)-enriched feed, and (D) *n*-non-anoic acid (C9)-enriched feed**. The fractions were assigned to groups a–w on the basis of their ghrelin activity. Ghrelin activity in the equivalent of 400 mg stomach tissue is expressed in terms of fluorescence change in CHO cells expressing rat GHS-R1a The maximum changes in fluorescence are plotted.

By means of cDNA cloning, we isolated four different barfin flounder ghrelin cDNAs (Figure 1): (1) a 948-bp cDNA that encodes a 106-aa prepro-ghrelin (acc. no. AB823534, Figure 1A); (2) a 945-bp cDNA that encodes des-Q65 prepro-ghrelin, which has 105 aa and lacks the 65th glutamine of the 106-aa prepro-ghrelin (acc. no. AB824842, Figure 1A); (3) a 557-bp cDNA that encodes a 106-aa prepro-ghrelin but lacks part of the 3′-untranslated region of AB823534 (acc. no. AB824843, Figure 1B), and (4) a 554-bp cDNA that encodes des-Q65 prepro-ghrelin but lacks part of the 3′-untranslated region of AB823534 (acc. no. AB824844, Figure 1B). These cDNAs encoded identical mature ghrelins.

On the basis of sequence homology with the ghrelins of other fish, the barfin flounder ghrelin was predicted to be a 20-aa peptide (GSSFLSPSHKPPNKGKPPRA) with a C-terminal amide structure. Phylogenetic analysis revealed that the barfin flounder ghrelin belongs to the evolutionarily most advanced group of Teleostei, which includes Perciformes and Gasterosteiformes (Figure 2), and shows the highest identity (93.4%) to Atlantic halibut ghrelin.

### Identification of ghrelin in the stomach of fish fed a normal feed

Fractions obtained by means of CM ion-exchange HPLC of stomach extracts of barfin flounder fed a normal feed were divided into seven groups (a–g) on the basis of their ghrelin activity (Figure 3A). The group “e” fractions were subjected to preparative reverse-phase HPLC on a symmetry column (Figure 3B) and then to reverse-phase HPLC on a diphenyl column (Figure 3C) to afford peptide P1, which was the peptide obtained in the highest yield. The peptides obtained in the second (P2) and the third and fourth highest yields (P3 and P4, respectively) were isolated from the group “g” and group “c” fractions, respectively (Figures 3D,E, respectively).

The amino acid sequence of peptide P1 was determined to be GSXFLSPSHKPPNKGKP (X was not detected). This sequence corresponded to 17 of the amino acid residues in the 20-aa sequence of the barfin flounder ghrelin (GSSFLSPSHKPPNKGKP^17^PRA) deduced from cDNA. Thus, we concluded that the isolated peptide was the barfin flounder ghrelin and that the undetected third aa was a serine. Subsequent mass spectrometric analysis of peptide P1 revealed that its molecular weight [M + H]^+^ was 2172.06, and the molecular form corresponded to decanoylated ghrelin with 19 aas [GSS(C10:0)FLSPSHKPPNKGKPPR]; the last alanine from the 20-aa peptide was missing. This result indicates that the major form of ghrelin isolated from the barfin flounder stomach extracts was slightly different from the form deduced from the cDNA sequence. The molecular forms of the other peptides isolated from in the stomach of fish fed a normal feed (Cont) are summarized in Table 1. Peptide P2 was determined to be a C-terminal amidated 20-aa peptide with C10 modification, and P3 and P4 were determined to be, respectively, a C8-modified 19-aa peptide and an adduct of a C8-modified 19-aa peptide and sodium-like ion with a 24 *m*/*z*.

**Table 1:** **Masses and expected molecular forms of ghrelins purified from barfin flounder stomach extracts by carboxymethyl ion-exchange HPLC**.

Feed group^1^	HPLC fraction group (elution time, min)^2^	Actual mass [M + H]^+^	Expected ghrelin form	Abundance^3^
Cont	c (33–37)	2144.12	19-(C8:0)	Δ
		2272.17	21-(C8:0)	
		2168.03	19-(C8:0) + 24	Δ
		2296.07	21-(C8:0) + 24	
	d (38–40)	2158.04	19-(C9:0)	
		2170.04	19-(C10:1)	
		2286.09	21-(C9:0)	
		2298.09	21-(C10:1)	
		2170.04	19-(C10:1)	
		2298.09	21-(C10:1)	
	e (43–46)	2214.19	20-(C8:0)-amide	
		2238.10	20-(C10:2)-amide	
		2172.06	19-(C10:0)	⊚
	f (49–50)	2228.09	20-(C9:0)-amide	
		2240.09	20-(C10:1)-amide	
		2240.10	20-(C10:1)-amide	
	g (55–56)	2242.12	20-(C10:0)-amide	○
C7	h (35–37)	2144.02	19-(C8:0)	
		2188.05	19-(C10:1) + 17	
	i (38–40)	2170.03	19-(C10:1)	△
		2298.07	21-(C10:1)	
	j (42–45)	2300.08	21-(C10:0)	○
	k (45–47)	2172.04	19-(C10:0)	⊚
	l (48–50)	2240.09	20-(C10:1)-amide	
	m (55–56)	2242.09	21-(C10:0)-amide	○
C8	o (33–37)	2146.02	19-(C8:0)	⊚
		2272.06	21-(C8:0)	
	p (39–41)	2157.98	19-(C9:0)	
		2170.03	19-(C10:1)	
		2170.03	19-(C10:1)	
	q (43–46)	2242.09	20-(C10:0)-amide	△
	r (55–56)	2214.07	20-(C8:0)-amide	○
		2172.03	19-(C10:0)	○
C9	s (30–31)	2159.02	19-(C9:0) + 1	
		2001.95	18-(C9:0)	
	t (35–37)	2144.02	19-(C8:0)	
		2168.02	19-(C10:2)	
		2286.09	21-(C9:0)	△
	u (38–41)	2158.03	19-(C9:0)	⊚
	v (45–46)	2172.04	19-(C10:0)	○
	w (49–50)	2228.07	20-(C9:0)-amide	○

^1^*Cont, normal feed; C7, feed enriched with n-heptanoic acid; C8, feed enriched with n-octanoic acid; C9, feed enriched with n-non-anoic acid*.

^2^*Carboxymethyl ion-exchange HPLC fractions were grouped on the basis of ghrelin activity*.

^3^*Fractions containing the peptides obtained in the (⊚) highest, (○) second highest, and (△) third highest yields*.

### Determination of molecular form of ghrelin in the stomach of fish fed fatty acid-containing feed

We found that gastric expression of ghrelin in fish fed fatty acid-containing feed was not significantly different from expression in control fish that were fed normal feed; the mean mRNA copy numbers × 10^4^ ± SEMs were as follows: controls, 6.57 ± 2.91 (*n* = 4); C7-enriched feed, 3.31 ± 0.77 (*n* = 4); C8-enriched feed, 4.29 ± 0.91 (*n* = 6); C9-enriched feed, 5.17 ± 1.48 (*n* = 6).

We compared the distributions of ghrelin activity in CM ion-exchange HPLC fractions obtained from fish that were fed C7-, C8-, and C9-enriched feeds (Figure 4). Fish that ingested C7 showed lower total ghrelin activity (Figure 4B) than control fish (Figure 4A), but the two distribution profiles differed only slightly. In contrast, in fish that ingested C8, ghrelin activities of the fractions that eluted from 33 to 36 min (group o) and from 43 to 46 min (group q) were much higher than activities of the corresponding fractions for the control fish, whereas the activities of the fractions that eluted from 55 to 56 min (group r) were lower (Figure 4C). The greatest change in the distribution profile was observed for fish that ingested C9 (Figure 4D); the ghrelin activities in the fractions that eluted from 30 to 31 min (group s), from 38 to 41 min (group u), and from 49 to 50 min (group w) were substantially higher than the activities for the controls, but activities in the fractions that eluted from 35 to 37 min (group t) and from 45 to 46 min (group v) were lower than the activities for the controls.

Table 1 summarizes the molecular weights and expected forms of the ghrelin peptides isolated from the groups of fractions shown in Figure 4. From the fish that ingested the C7-enriched feed, the peptide obtained in the highest yield was a 19-aa peptide with a C10 modification, as was the case for the control fish. The purified peptide eluted from 45 to 47 min (group k), which was nearly the same as the elution time for the peptide obtained in the highest yield from the control fish (group e). We looked for different groups, but were unable to identify a peptide modified by C7. From the fish that ingested the C8-enriched feed, the most abundant peptide was a 19-aa peptide with a C8 modification, which was isolated from fractions that eluted from 33 to 37 min (group o). From the fish that ingested the C9-enriched feed, the most abundant peptide was a 19-aa peptide with a C9 modification, which was isolated from fractions that eluted from 38 to 41 min (group u).

### Distribution of ghrelin activity in fractions obtained from stomach extracts of control fish and fish fed fatty acid-containing feed

**Figure 5:**
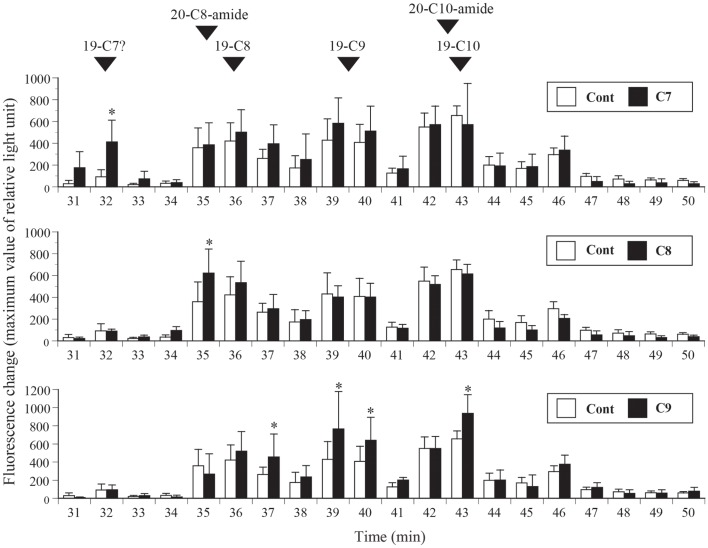
**Distribution of ghrelin activity in fractions separated by reverse-phase HPLC**. Ghrelin activity in the equivalent of 100 mg stomach tissue is expressed in terms of fluorescence change in CHO cells expressing rat GHS-R1a. The maximum changes in fluorescence are plotted. The numbers of samples used are as follows: control (Cont, *n* = 4); *n*-heptanoic acid (C7)-enriched feed (*n* = 4); *n*-octanoic acid (C8)-enriched feed (*n* = 6); and *n*-non-anoic acid (C9)-enriched feed (*n* = 6). The values for each group were compared to the control values by analysis of variance followed by Fisher’s protected least significant difference test. Differences were considered significant at *P* < 0.05. The elution times of isolated native barfin flounder ghrelin, C8-ghrelin with 19 amino acids (aas) (19-C8), C9-ghrelin with 19 aas (19-C9), C10-ghrelin with 19 aas (19-C10), C8-ghrelin with 20 aas and C-terminal amidation (20-C8-amide), and C10-ghrelin with 20 aas and C-terminal amidation (20-C10-amide) are indicated by arrowheads. The expected elution time of C7-ghrelin with 19 aas (19-C7) is also indicated; the time is based on the results for 19-C8, 19-C9, and 19-C10.

In this experiment, stomach extracts (equivalent to 100 mg of tissue) from control fish or fish fed fatty acid-enriched feed was subjected to reverse-phase HPLC only, without further purification, and ghrelin activities in the separated fractions were compared (Figure 5). Ghrelin activities were significantly higher in the 32-min fraction of the C7-enriched group, the 35-min fraction of the C8-enriched group, and the 37-, 39-, 40-, and 43-min fractions of the C9-enriched group compared to the activities in the corresponding fractions of the control fish. These elution times correspond to the elution time of the most abundant molecular form of ghrelin isolated from each group.

## Discussion

We determined the nucleotide sequence of ghrelin cDNA in the barfin flounder for the first time. From the cDNA sequence, the mature peptide was predicted to be a 20-aa peptide (GSSFLSPSHKPPNKGKPPRA) with an amide structure at the C-terminus, and the expected 20-aa peptide was in fact isolated from the stomach extracts of fish fed a normal diet. However, the peptide isolated in the highest yield was a 19-aa peptide (GSSFLSPSHKPPNKGKPPR) lacking the last alanine. This result is similar to results reported for goldfish ghrelin (20). A possible mechanism for the lack of alanine is due to an effect of arginine endopeptidase, trypsin, for the proteolytic processing. However, the detail mechanisms are unclear.

The 19-aa ghrelin was modified not with C8, which is the usual acyl modification of ghrelin, but with C10 (1). This result is not completely unprecedented: C10-modified ghrelin has occasionally been identified in non-mammalian vertebrates (14–17, 19, 21), and a C10-modified ghrelin is the major form in Mozambique tilapia (18). C10-modified ghrelin is also present in mammals (25, 26). In this study, we also detected ghrelin peptides acylated with C8, C9, and unsaturated decanoic acids (C10:1 and C10:2). Modification of ghrelin with various fatty acids has been known in other fish and animals not having been limited to barfin flounder. In the barfin flounder, however, C9 appears to be a substrate for ghrelin acylation, even though acylation with C9 is rare in other vertebrates, except cats and goats (27, 28).

In previous studies of neonatal chicks that had not yet begun to eat feed, the expression of ghrelin mRNA was detected, while the production of acylated ghrelin was not (24, 29). This result strongly suggests that expression of the ghrelin gene is unaffected by feeding, and that fatty acids (substrates) in feed are involved in ghrelin acylation. In the present study, we found that expression of the ghrelin gene was unaffected by the addition of fatty acids to feed. Similar results have been reported in mice (9) and chickens (24). It is likely that intake of fatty acid does not affect the gastric expression of the ghrelin gene in vertebrates.

Different fatty acids cause alteration in the ghrelin protein levels of the stomach as observed by analyses used reverse-phase HPLC or ion-exchange HPLC of stomach extracts (Figures 4 and 5), and the use of different fatty acids in the feed resulted in the identification of different ghrelin molecules (Table 1). The present study showed that in fish given feed containing C8 or C9, ghrelins modified with these fatty acids were the major form in the stomach. In contrast, C10-ghrelin was the major form in fish fed normal feed. This result clearly indicates that dietary fatty acids were substrates for ghrelin acylation in fish, as is the case for mice and chickens.

In a previous study by Nishi et al. (9), *n*-heptanoyl ghrelin, an unnatural form of ghrelin, has been isolated in the stomach of mice after ingestion of either *n*-heptanoic acid or glyceryl triheptanoate. In the present study, ghrelin modified by C7 did not identify in the stomach of fish fed control diet, indicating that C7-ghrelin is an unnatural form. In this study, C7-enriched diet was given to barfin flounder, and all the fish ate the same amount of food. However, we could not isolate ghrelin modified with C7. A possible reason is that hat there was not enough C7-ghrelin content to isolate. Meanwhile, C7-ghrelin-like activity could be detected in the analysis of the stomach extract ingested C7 that used reverse-phase HPLC. The reason of the success is probably that this analysis was not subjected to any purification processes other than reverse-phase HPLC, and it prevented a loss of peptide in the purification processes. The result detected an unnatural form C7-ghrelin in this study further supports that dietary fatty acids were substrates for ghrelin acylation in fish. In this study, C9-ghrelin was purified not only in fish fed C9-enriched feed but also in fish fed normal feed. As described earlier, ghrelin acylation with C9 is rare in other vertebrates. Therefore, this result observed in the barfin flounder came from the reason that C9 could be basically used for ghrelin acylation.

It would be helpful if the effect of the diets with increasing the chain length of fatty acids (e.g., C16 and C18) on acylation of ghrelin was tested. We did not perform such an experiment in this study. Nishi et al. (9) observed that neither *n*-butyryl (C4) nor *n*-palmitoyl (C16) ghrelin is detected when mice are given short-chain triacylglyceride glyceryl tributyrate or glyceryl tripalmitate, suggesting short- or long-chain fatty acids are not used for ghrelin acylation. Ohgusu et al. (30) used recombinant GOAT and demonstrated that GOAT did not modify des-acyl ghrelin with long-chain fatty acids such as *n*-palmitoyl-CoA, and *n*-myristoyl-CoA *in vitro*. This result also shows that middle-chain fatty acids derived from the degradation of C16 or C18 fatty acids do not involve in ghrelin acylation.

In CM ion-exchange HPLC, we observed that when the ghrelin activity in one fraction increased, the activity in other fractions decreased. For example, we observed a substantial difference between the distribution of ghrelin activity in fish that ingested C9 (Figure 4D) and the distribution in control fish (Figure 4A). Similar results have been observed in mice: ingestion of C6:0-medium-chain triacylglycerols increases the amount of C6-ghrelin but decreases the amount of C8-ghrelin (9). This result suggests that GOAT has a finite catalytic capacity. In addition, we observed that total ghrelin activity decreased when C7 was given to fish, suggesting that C7 might inhibit enzymatic activity.

Currently, the only fish for which the GOAT sequence is available is the zebrafish. Shlimun and Unniappan (11) have indicated which aa are essential for the catalytic activity of GOAT in mammals and fish. Ohgusu et al. (30) reported that the four aa at the N-terminal of ghrelin (GSSF) constitute the minimum core motif required for substrate recognition of GOAT. Because this core sequence is conserved in the ghrelin of the barfin flounder, it is likely that a GOAT-like enzyme plays a role in ghrelin acylation, although the detailed mechanisms remain to be elucidated.

In summary, we determined for the first time the primary structure of ghrelin peptides in the barfin flounder and found that ingestion of various fatty acids affected ghrelin acylation in these fish, as is the case in mice and chickens. This result indicates that the mechanism by which fatty acids in foods are used for post-transcriptional modification of ghrelin peptides and the mechanism by which modification regulates the biological activity of the peptide are conserved between fish and mammals. The possession of this post-transcriptional mechanism in vertebrates may explain why they were able to acquire ingenious and complicated processes for strict regulation of lipids and glucose.

## Conflict of Interest Statement

The authors declare that the research was conducted in the absence of any commercial or financial relationships that could be construed as a potential conflict of interest.

## References

[1] Kojima M Hosoda H Date Y Nakazato M Matuso H Kangawa K Ghrelin is a growth-hormone-releasing acylated peptide. Nature (1999) 402:656–60 10.1038/45230 10604470

[2] Kaiya H Kojima M Hosoda H Koda A Yamamoto K Kitajima Y Bullfrog ghrelin is modified by *n*-octanoic acid at its third threonine residue. J Biol Chem (2001) 276:40441–8 10.1074/jbc.M105212200 11546772

[3] Gutierrez JA Solenberg PJ Perkins DR Willency JA Knierman MD Jin Z Ghrelin octanoylation mediated by an orphan lipid transferase. Proc Natl Acad Sci U S A (2008) 105:6320–5 10.1073/pnas.0800708105 18443287 PMC2359796

[4] Yang J Brown MS Liang G Grishin NV Goldstein JL Identification of the acyltransferase that octanoylates ghrelin, an appetite-stimulating peptide hormone. Cell (2008) 132:387–96 10.1016/j.cell.2008.01.017 18267071

[5] Sakata I Yang J Lee CE Osborne-Lawrence S Rovinsky SA Elmquist JK Colocalization of ghrelin *O*-acyltransferase and ghrelin in gastric mucosal cells. Am J Physiol Endocrinol Metab (2009) 297:E134–41 10.1152/ajpendo.90859.2008 19401456 PMC2711663

[6] Kang K Zmuda E Sleeman MW Physiological role of ghrelin as revealed by the ghrelin and GOAT knockout mice. Peptides (2011) 32:2236–41 10.1016/j.peptides.2011.04.028 21600256

[7] Kojima M Ida T Sato T Structure of mammalian and nonmammalian ghrelins. Vitam Horm (2008) 77:31–46 10.1016/S0083-6729(06)77003-0 17983852

[8] Hosoda H Kojima M Mizushima T Shimizu S Kangawa K Structural divergence of human ghrelin. Identification of multiple ghrelin-derived molecules produced by post-translational processing. J Biol Chem (2003) 278:64–70 10.1074/jbc.M205366200 12414809

[9] Nishi Y Hiejima H Hosoda H Kaiya H Mori K Fukue Y Ingested medium-chain fatty acids are directly utilized for the acyl modification of ghrelin. Endocrinology (2005) 146:2255–64 10.1210/en.2004-0695 15677766

[10] Kirchner H Gutierrez JA Solenberg PJ Pfluger PT Czyzyk TA Willency JA GOAT links dietary lipids with the endocrine control of energy balance. Nat Med (2009) 15:741–5 10.1038/nm.1997 19503064 PMC2789701

[11] Shlimun A Unniappan S Ghrelin *O*-acyl transferase: bridging ghrelin and energy homeostasis. Int J Pept (2011) 2011:217957 10.1155/2011/217957 21941572 PMC3175403

[12] Kaiya H Miyazato M Kangawa K Peter RE Unniappan S Ghrelin: a multifunctional hormone in non-mammalian vertebrates. Comp Biochem Physiol A Mol Integr Physiol (2008) 149:109–28 10.1016/j.cbpa.2007.12.004 18222718

[13] Kaiya H Miyazato M Kangawa K Recent advances in the phylogenetic study of ghrelin. Peptides (2011) 32:2155–74 10.1016/j.peptides.2011.04.027 21600258

[14] Kaiya H Van der Gayten S Kojima M Hosoda H Kitajima Y Matsumoto M Chicken ghrelin: purification, cDNA cloning, and biological activity. Endocrinology (2002) 143:3445–63 10.1210/en.2002-220255 12193558

[15] Kaiya H Sakata I Kojima M Hosoda H Sakai T Kangawa K Structural determination and histochemical localization of ghrelin in the red-eared slider turtle, *Trachemys scripta elegans*. Gen Comp Endocrinol (2004) 138:50–7 10.1016/j.ygcen.2004.05.005 15242751

[16] Kaiya H Kojima M Hosoda H Moriyama S Takahashi A Kawauchi H Peptide purification, cDNA and genomic DNA cloning, and functional characterization of ghrelin in rainbow trout. Endocrinology (2003) 144:5215–26 10.1210/en.2003-1085 12970156

[17] Kaiya H Kojima M Hosoda H Riley LG Hirano T Grau EG Amidated fish ghrelin: purification, cDNA cloning in the Japanese eel and its biological activity. J Endocrinol (2003) 176:415–23 10.1677/joe.0.1760415 12630926

[18] Kaiya H Kojima M Hosoda H Riley LG Hirano T Grau EG Identification of tilapia ghrelin and its effects on growth hormone and prolactin release in the tilapia, *Oreochromis mossambicus*. Comp Biochem Physiol B Biochem Mol Biol (2003) 135:421–9 10.1016/S1096-4959(03)00109-X 12831762

[19] Kaiya H Small BC Lelania Bilodeau A Shepherd BS Kojima M Hosoda H Purification, cDNA cloning, and characterization of ghrelin in channel catfish, *Ictalurus punctatus*. Gen Comp Endocrinol (2005) 143:201–10 10.1016/j.ygcen.2005.03.012 16111526

[20] Miura T Maruyama K Kaiya H Miyazato M Kangawa K Uchiyama M Purification and properties of ghrelin from the intestine of the goldfish, *Carassius auratus*. Peptides (2009) 30:758–65 10.1016/j.peptides.2008.12.016 19150635

[21] Kawakoshi A Kaiya H Riley LG Hirano T Grau EG Miyazato M Identification of a ghrelin-like peptide in two species of shark, *Sphyrna lewini* and *Carcharhinus melanopterus*. Gen Comp Endocrinol (2007) 151:259–68 10.1016/j.ygcen.2006.10.012 17362948

[22] Fox BK Riley LG Dorough C Kaiya H Hirano T Grau EG Effects of homologous ghrelins on the growth hormone/insulin-like growth factor-I in the tilapia, *Oreochromis mossambicus*. Zoolog Sci (2007) 24:391–400 10.2108/zsj.24.391 17867837

[23] Schwandt SE Peddu SC Riley LG Differential roles for octanoylated and decanoylated ghrelins in regulating appetite and metabolism. Int J Pept (2010) 2010:275804 20700399 10.1155/2010/275804 PMC2911580

[24] Yamato M Sakata I Wada R Kaiya H Sakai T Exogenous administration of octanoic acid accelerates octanoylated ghrelin production in the proventriculus of neonatal chicks. Biochem Biophys Res Commun (2005) 333:583–9 10.1016/j.bbrc.2005.05.107 15953586

[25] Nishi Y Yoh J Hiejima H Kojima M Structures and molecular forms of the ghrelin-family peptides. Peptides (2011) 32:2175–82 10.1016/j.peptides.2011.07.024 21839128

[26] Yoh J Nishi Y Hosoda H Tajiri Y Yamada K Yanase T Plasma levels of *n*-decanoyl ghrelin, another acyl- and active-form of ghrelin, in human subjects and the effect of glucose- or meal-ingestion on its dynamics. Regul Pept (2011) 167:140–8 10.1016/j.regpep.2010.12.010 21237214

[27] Ida T Miyazato M Naganobu K Nakahara K Sato M Lin XZ Purification and characterization of feline ghrelin and its possible role. Domest Anim Endocrinol (2007) 32:93–105 10.1016/j.domaniend.2006.01.002 16466902

[28] Ida T Miyazato M Lin XZ Kaiya H Sato T Nakahara K Purification and characterization of caprine ghrelin and its effect on growth hormone release. J Mol Neurosci (2010) 42:99–105 10.1007/s12031-010-9379-0 20437258

[29] Wada R Sakata I Kaiya H Nakamura K Hayashi Y Kangawa K Existence of ghrelin-immunopositive and -expressing cells in the proventriculus of the hatching and adult chicken. Regul Pept (2003) 111:123–8 10.1016/S0167-0115(02)00265-3 12609759

[30] Ohgusu H Shirouzu K Nakamura Y Nakashima Y Ida T Sato T Ghrelin *O*-acyltransferase (GOAT) has a preference for n-hexanoyl-CoA over *n*-octanoyl-CoA as an acyl donor. Biochem Biophys Res Commun (2009) 386:153–8 10.1016/j.bbrc.2009.06.001 19501572

